# *In vivo* MRI and PET imaging in a translational ILD mouse model expressing non-resolving fibrosis and bronchiectasis-like pathology after repeated systemic exposure to bleomycin

**DOI:** 10.3389/fmed.2024.1276420

**Published:** 2024-04-09

**Authors:** Irma Mahmutovic Persson, Nina Fransén Petterson, Jian Liu, René in ‘t Zandt, Carla Carvalho, Anders Örbom, Lars E. Olsson, Karin von Wachenfeldt

**Affiliations:** ^1^Medical Radiation Physics, Institution of Translational Medicine, Lund University, Malmö, Sweden; ^2^Lund University BioImaging Centre (LBIC), Medical Faculty, Lund University, Lund, Sweden; ^3^Truly Labs, Lund, Sweden; ^4^Division of Oncology, Department of Clinical Sciences Lund, Lund University, Lund, Sweden; ^5^Department of Hematology, Oncology, and Radiation Physics, Skåne University Hospital, Malmö, Sweden

**Keywords:** longitudinal imaging, chronic model, lung toxicity/lung injury, drug-induced interstitial lung disease, fibrosis, animal models/mouse models, positron emission tomography, magnetic resonance imaging

## Abstract

Drug-induced interstitial lung disease (ILD) is crucial to detect early to achieve the best treatment outcome. Optimally, non-invasive imaging biomarkers can be used for early detection of disease progression and treatment follow-up. Therefore, reliable *in vivo* models are warranted in new imaging biomarker development to accelerate better-targeted treatment options. Single-dose bleomycin models have, for a long time, served as a reference model in fibrosis and lung injury research. Here, we aimed to use a clinically more relevant animal model by systemic exposure to bleomycin and assessing disease progression over time by combined magnetic resonance imaging (MRI) and positron emission tomography (PET) imaging.

**Methods:**

C57BL/6 mice received bleomycin (i.p. 35iU/kg) or saline as control twice per week for 4 weeks. Mice were monitored until 2 weeks after cessation of bleomycin administration (w4 + 1 and w4 + 2), referred to as the resting period. MRI scans were performed in weeks 3 and 4 and during the resting weeks. [^18^F]FDG-PET was performed at the last week of dosing (w4) and 2 weeks after the last dosing (w4 + 2). Lung tissue sections were stained with Masson’s trichrome and evaluated by modified Ashcroft scoring. Lung volume and lesion volumes were assessed using MRI, as well as 3D mapping of the central airways.

**Results and discussion:**

Bleomycin-challenged mice showed increased lung weights (*p* < 0.05), while total lung volume was unchanged (w4 and onward). Histology analysis demonstrated fibrotic lesions emanating from the distal parts of the lung. Fibrosis progression was visualized by MRI with significantly increased high signal in bleomycin-exposed lungs compared to controls (*p* < 0.05). In addition, a significant increase in central airway diameter (*p* < 0.01) was displayed in bleomycin-exposed animals compared to controls and further continued to dilate as the disease progressed, comparing the bleomycin groups over time (*p* < 0.05–0.001). Lung [^18^F]FDG uptake was significantly elevated in bleomycin-exposed mice compared to controls (*p* < 0.05).

**Conclusion:**

Non-invasive imaging displayed progressing lesions in the lungs of bleomycin-exposed mice, using two distinct MRI sequences and [^18^F]FDG-PET. With observed fibrosis progression emanating from distal lung areas, dilation of the central airways was evident. Taken together, this chronic bleomycin-exposure model is translationally more relevant for studying lung injury in ILD and particularly in the context of DIILD.

## Introduction

Interstitial lung disease (ILD) is a heterogeneous group of pulmonary parenchymal diseases characterized by varying degrees of inflammation and fibrosis in the lung interstitium. ILD pathologies present with a wide range of symptoms and can be classified into different subgroups. The ILD subgroup of known causes includes external and environmentally induced disease due to silica or asbestos exposure. Drug-induced ILD (DIILD) is another example belonging to this ILD subgroup, where certain medications can induce side effects, initiating inflammation in the lung with a potentially progressive and irreversible fibrotic response (1–3). To date, there are more than 350 drugs on the market that have been associated with DIILD, and this number is predicted to increase over the coming decades (2, 4–6). DIILD is difficult to diagnose since there are no clear diagnostic traits. Different drugs cause varying histopathological patterns, not accounting for patient variability (2, 7, 8). DIILD is therefore thought to be an underdiagnosed condition (5, 9, 10). The initial onset of DIILD can manifest as various types of inflammatory patterns, including cellular infiltrates and sometimes with the presence of granulomas. Different drug classes are associated with DIILD and implicated in the induction of lung disease at various rates (2, 11), and cytotoxic drugs, in general, are known to induce severe side effects. In particular, bleomycin, an agent mainly used for the treatment of testicular cancer and Hodgkin’s lymphoma, has been implicated in inducing ILD in up to 21% of all patients taking the drug (2, 7, 10, 12, 13).

To identify patients developing DIILD at an early stage, as well as to be able to follow disease progression or regression, biomarkers such as non-invasive imaging biomarkers would serve as important tools (14–16). Currently, the main imaging modalities for diagnosis of ILD are chest X-ray and high-resolution computed tomography (HRCT). However, modalities such as magnetic resonance imaging (MRI) and positron emission tomography (PET) are being increasingly employed in the clinic for lung imaging and for longitudinal disease assessment (14, 17–19).

In the search for new imaging biomarkers in lung injury and DIILD, animal models exhibiting key aspects of human disease are warranted. Such a model should optimally mimic the typical clinical scenario, i.e., systemic and prolonged exposure of the agent causing the disease. To date, various lung injury models exist, with the primary goal of studying fibrosis, while other models are investigating the inflammatory aspects. However, the most common animal model used for studying ILD associated with lung fibrosis is the acute administration of a single dose of bleomycin, instilled via the intratracheal route (20, 21), which does not mimic human exposure or pathophysiology very well. This induction regime generates an initial inflammation, which can either progress into fibrosis or spontaneously resolve and is associated with an increase in the total lung volume (21, 22). The disease progression and appearance in such models do not fully resemble human DIILD, as they display an initial high level of inflammation, mainly comprising of lesions that appear in the central airways. In patients developing DIILD, the drug-inducing lung toxicity is normally administrated systemically, by intravenous or subcutaneous injection, or as orally given medications. Thus, the drug commonly reaches the lung via the circulation. This results in a different disease pattern characterized by chronic inflammation and fibrosis development over time, originating from the peripheral lung parenchyma (2, 10, 23, 24). In addition to disease induction being introduced systemically, another important aspect is to generate a model where the injury persists after the inducing stimuli have been removed and exposure stopped. This scenario would present the optimal mimic of clinical DIILD with disease continuation, or even worsening, after drug withdrawal. Examples of persistent lung injury models are few, but silica or asbestos have been employed as pro-fibrotic substances to induce non-resolving lung injury in rodents (22, 25, 26). In these models, the generated fibrosis does not resolve but progresses over time, as demonstrated by imaging performed for disease assessment (25). Repeated exposures of bleomycin given via other administration routes than the commonly applied local approach have previously been used to create lung injury by either subcutaneous injections or oropharyngeal aspiration regime, some also including disease assessment by imaging (27–30). Consequently, to study DIILD and investigate non-invasive imaging biomarkers for early and specific disease detection, translational ILD models with longitudinal imaging are needed.

In the current study, we performed longitudinal multimodal MRI and PET imaging on animals systemically exposed to bleomycin, both during disease progression and after bleomycin treatment had stopped. Performing longitudinal imaging in this model generated further valuable knowledge in terms of disease model characterization and subsequent disease understanding. Our aim with the current study was to refine and characterize a chronic model of ILD with repeated systemic exposure to bleomycin. Using non-invasive multimodal imaging techniques combining MRI and PET, we were able to follow the dynamic disease development by mapping the lesions representing both ongoing inflammation and fibrogenesis. Using both modalities as one multimodal technique provided complementary information in spatial and dynamic assessment of the inflammatory and fibrotic lesions, where the PET tracer Fludeoxyglucose [^18^F]FDG was employed for mapping areas of increased metabolism as an indication of ongoing disease and MRI to identify disease areas and structural alterations.

## Materials and methods

### Animal handling

All animal experiments were performed according to the protocols evaluated and approved by the local ethical committee in Lund/Malmö, Sweden, with permit numbers 4003/2017 and 3226/2017, and reported according to the ARRIVE guidelines (31). Animals were housed at Lund University and Medicon Village animal facilities with 12 h light/dark hours. A total of 53 male mice from the C57BL/6 strain were used in this study, and experiments were initiated when the mice were 8 weeks of age (24 g ± 1 g). Before the start of the study, the mice were allowed to acclimatize to the housing conditions for a minimum of 5 days. All animals had free access to water and food, and soft, wet food was provided inside the cage to animals receiving bleomycin to prevent substantial weight loss. If an animal showed signs of distress and in parallel severe body weight loss, and no signs of recovery were indicated within the following 2 days, they were removed from the study by intraperitoneal (i.p.) overdose injection of Pentobarbital Sodium (Apotek Produktion & Laboratorier AB, Sweden) and were not included in the final data points. In total, n = 12 bleomycin-exposed mice were removed from the study due to reaching humane endpoints or because they were deceased during their final scan while being anesthetized.

### Experimental set-up—induction of lung injury by repeated systemic bleomycin exposures

**Figure 1:**
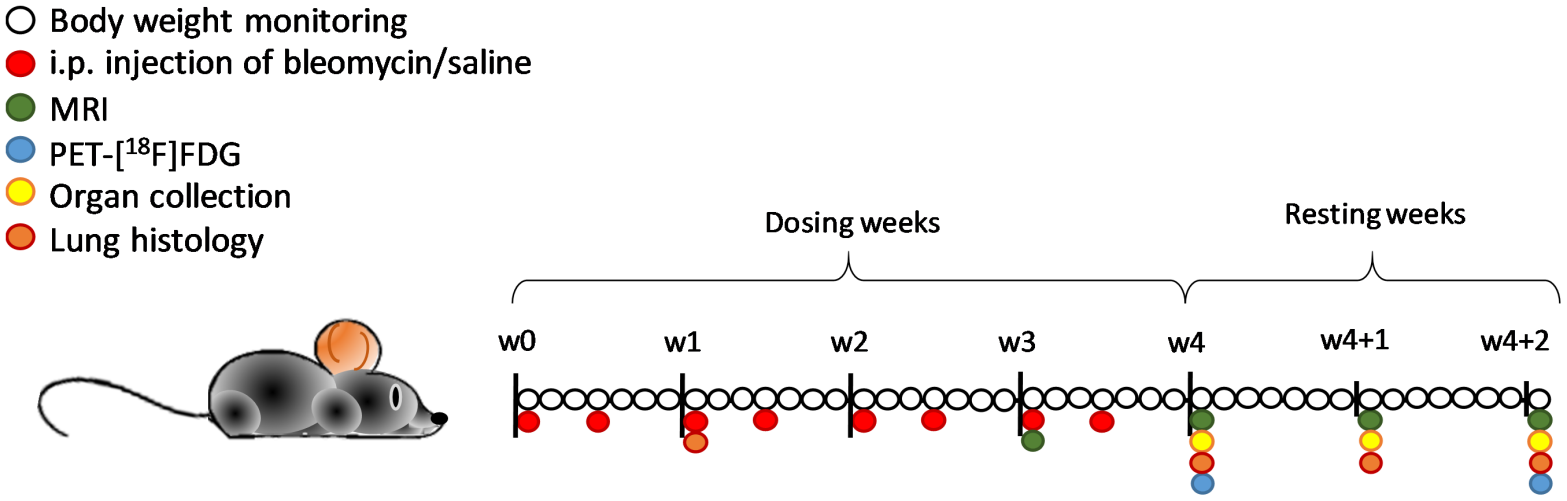
Schematic representation of the study layout with the imaging sessions and terminal endpoints in C57BL/6 mice. Daily body weight monitoring was done during 4 weeks of bleomycin dosing (two times per week) and an additional 2 weeks of resting period that followed. Imaging sessions were performed in w3 and onward, with MRI scanning every week from then, and PET imaging at w4 and w4 + 2. Terminal samples and organs were collected at the time points w1, w4, w4 + 1, and w4 + 2. In total, *n* = 12 mice were removed from the study due to humane endpoints or acute death (out of the total *n* = 53).

Bleomycin (Baxter, Apotek Produktion & Laboratorier AB, Sweden) was diluted in saline and administered at a concentration of 35iU per gram body weight. All mice were injected i.p. twice per week for 4 weeks. The control group received injections with the same volume of saline (vehicle) and are referred to as controls. Body weights were monitored daily, and the bleomycin dose was adjusted according to the body weights. After the final i.p. challenge, animals were followed for two additional resting weeks (w4 + 1 and w4 + 2). Imaging was performed at time points w3, w4, w4 + 1, and w4 + 2 (Figure 1) by either MRI only or in combination with PET/CT. At termination, the mice received an i.p. overdose of Pentobarbital Sodium, and subsequently, the left lung was processed for histology, and the lung lobes of the right side were dissected, weighed, and snap frozen. Terminal samples were collected at time points w1, w4, w4 + 1, and w4 + 2.

### *In vivo* multimodality imaging set-up

*In vivo* imaging was performed at Lund University BioImaging Centre (LBIC), combining multimodality imaging with MRI and PET/CT. The workflow was designed accordingly: intravenous PET tracer injection into the tail vein of mice placed in a restrainer. Directly after tracer injection, the animal was anesthetized with isoflurane and placed inside the Minerve animal bed (Équipement Vétérinaire Minerve, Esternay, France). Breathing sensor and temperature probe were put in place, and MRI scans were initiated, followed by PET/CT. All mice were placed in a supine position and anesthetized with isoflurane (IsoFlo vetOrion Pharma, Sweden) mixed with nitrous oxide and oxygen (mixture 1:1), delivered via the nose cone at approximately 1–2% during the image acquisition. The respiratory rate was monitored using a pneumatic pillow (SA instruments, NY, United States) and maintained at 70–90 breaths per minute by regulating the level of isoflurane. The body temperature was measured using a rectal probe and was kept constant by adjustments to the heating pad temperature in the animal bed. All imaging sessions were performed on spontaneously breathing animals.

### Magnetic resonance imaging

MRI scans were acquired on a preclinical 9.4 T MRI Biospec AV III (Bruker) using Paravision 7.0.1 (Bruker). The mice were initially scanned by applying a low-resolution scan (Localizer) to confirm the optimal positioning of the animal. Thereafter, two different pulse sequences were applied: Rapid Acquisition with Relaxation Enhancement (RARE) followed by a 3D dual-echo Ultra-Short-Echo (UTE) sequence. The main settings for the respiration-triggered RARE were TE = 16.17 ms, TR = 6,000 ms, echo train length of 8, with 2 averages, the FOV was 25 × 25 mm^2^, matrix size of 160×160, with a slice thickness of 0.5 mm, including in total 40 slices covering the entire lung. The pixel resolution was 0.156 × 0.156 × 0.5 mm^3^ after reconstruction. For the non-triggered 3D-UTE data acquisition, 238 data points were acquired per spoke, a total of 115,432 spokes, TR = 6.5 ms, flip angle of 5 degrees, FOV was 30 × 30 × 60 mm^3^, with two different echo times of 8.1 μs (short UTE) and 1.1 ms (long UTE). The data were reconstructed using a matrix size of 192 × 192 × 192 resulting in a pixel size of 0.156 × 0.156 × 0.31 mm^3^.

The experimental time was approximately 30 min in total, including the connection of the various sensors (breathing and temperature) through the scan time and possible repositioning of the FOV for optimal adjustment.

### PET/CT imaging

PET imaging was used for the overall monitoring of increased cell and tissue metabolism by employing the [^18^F]FDG tracer. PET/CT was performed on a Mediso imaging system (nanoSCAN® PET/CT, Mediso, Hungary) at two separate time points in the study to capture pathological changes after the final i.p. challenge (w4) and during the later phase of the model (w4 + 2), during the resting period. The PET tracer was injected at a dose of 15 MBq (±5 MBq), dissolved in saline, in a total volume of 100 μL. In brief, at each scan session, the injected PET radiotracer was allowed to circulate systemically for about 1 h (partially during the MRI acquisition) before the PET imaging was initiated. Directly after MRI acquisition, an initial overview scan (scout-view scan) was performed before PET/CT imaging (the acquisition was about 2 min) to obtain the optimal position of the animal. After processing the scout-view image, the optimal FOV was adjusted to ensure coverage of the entire lungs, followed by a CT scan of 10 min acquisition time, and immediately thereafter PET imaging was done. The PET scan acquisition time was 20 min, initiated 1 h (±5 min) after tracer injection.

### Imaging data processing and analysis

MRI images were reconstructed using Paravision 7 and exported to DICOM format from the Bruker database, while the reconstruction of PET data was performed using the standard protocol of Maximum Likelihood Estimation Method (MLEM post-reconstruction protocol) using the Nucline v2.01 nanoscan software. The signal from the PET images was divided by the decay-corrected injected dose to each animal to calculate the fractional uptake, i.e., total activity uptake per mm^3^ and the total uptake in the whole lung, which is referred to as tracer uptake.

For image analysis, the software VivoQuant™ 2021 (inviCRO Imaging Services and Software VivoQuant, www.invicro.com) was employed. Initial segmentation of the lungs was performed using the images generated by an MRI sequence with a long UTE of 1.1 ms from images acquired in the axial plane with a slice thickness of 0.5 mm, in a total of 40 slices covering the entire mouse lung. The region of interest (ROI) in each slice was drawn semi-manually, using the “spline tool,” within the software, and was guided by the template manuals. The ROI did not include the heart or vessels with clear attachment to the heart. A thorough demonstration of the ROI selection of various slices within one animal scan can be observed in Supplementary Figure S1. Segments of the generated lung-ROI were overlaid with the images acquired on PET/CT using the VivoQuant tools Registration/Orientation. Registration was performed for the images acquired during the same imaging session from different modalities. Thereby, besides total lung volume assessment, additional data could be extracted using identical ROI from each scan session, with pixel intensity for the MRI and [^18^F]FDG signal uptake within the same lung region.

For lung lesion assessment by MRI, the pixel intensity values were exported as histograms from the RARE sequence scans and long UTE scans from each segmented lung-ROI. Thereafter, the histogram line-intersect method (32) was applied, and the threshold for “high-signal” of the total ROI was identified and plotted as “lesion area” mm^3^. MRI data from the short UTE sequence of 8.1 μs, with apparent visualization of lung tissue signal, allowed for more precise airway rendering and was therefore used to assess the central airways (Supplementary Figure S2A). The global thresholding tool in VivoQuant was used to assess the airway 3D rendering as described below. The threshold signal for air was extracted within the slice corresponding to where the trachea branches into the two main bronchi, and then the airway branching was possible to extract until the threshold signal changed. The extracted ROI of the central airways was then expressed as volume mm^3^. Further details on how the volume of the central airways was assessed are demonstrated in detail in Supplementary Figure S2B.

### Histological analysis

At termination, organs were collected for histological evaluation. The liver and kidneys were collected to assess potential systemic toxicity, fixed in 4% paraformaldehyde, and subsequently paraffin-embedded and sectioned before H&E staining (Histolab Products) and Picro Sirius Red staining (Abcam).

For the lung tissue processing, the left lung lobe was insufflated using 4% paraformaldehyde for adequate fixation by applying an even pressure corresponding to 15–20 cm H_2_O at a steady pace during insufflation and stored submerged in fixation overnight. Thereafter, the lungs were dehydrated, embedded in paraffin, and sectioned into 4 μm sections. The sectioning was performed in the sagittal plane, and the sections were obtained at two different positions in each left lobe. One position covered the large and central airways, while the second position covered the peripheral/distal parts, thus assessing the small airways.

The sections were stained by H&E, Picro Sirius Red, and Masson’s trichrome staining kit (Polysciences, Hirschberg an der Bergstrasse, Germany) for histopathological evaluation. Staining kits were used according to the manufacturer’s instructions, and all lung samples stained by Masson’s trichrome were subsequently assessed quantitatively using the modified Ashcroft scoring system (33). Scoring was performed by two independent and blinded observers for the two positions of each sample and presented as a total mean for that particular sample.

Furthermore, specific immunohistochemical staining was performed on the sectioned lung slides, targeting immune cell markers CD45 and CD11b for inflammation assessment. Sections were deparaffinized and rehydrated, following antigen retrieval, by heating the sections for 20 min in citrate buffer with pH 6. Endogenous peroxidase was blocked by incubation with 1% H_2_O_2_ for 15 min, and the sections were subsequently blocked using 5% normal mouse serum (NMS) for 30 min at room temperature. This was followed by incubation with primary antibodies: CD11b diluted 1:400 (Abcam, United Kingdom) and CD45 diluted 1:500 (Abcam, UK) in PBS with 5% NMS. Staining was visualized using anti-rabbit Bright Vision HRP (Immunologic, NL) and DAB. The slides were counterstained using Hematoxylin for 30 s, and then dehydrated and mounted in mounting media. The slides were left to dry overnight before analysis.

Immunohistochemistry stained slides were scanned (Zeiss Axio scan Z1 imager, Carl Zeiss microscopy, 246 GmbH, Germany) and evaluated by the software ImageJ. Fiji (v.2024) using the plug-in ImmunoRatio (v.2024). CD11b-positive cells were counted and presented as a ratio of total cells within each tissue section, according to a previously published protocol (34). The quantification was done at two positions from each lung, presented as a mean percentage, and then plotted as one value for each mouse. Further details are given in Supplementary Figure S3.

### Statistical analysis

All data were tested for statistically significant differences using GraphPadPrism version 9.03 (GraphPad Software, San Diego, California, United States, www.graphpad.com). The one-way ANOVA test was applied to identify differences between the groups, with *post-hoc* testing using Bonferroni’s multiple comparisons test. The student’s *t*-test or two-tailed Mann–Whitney test was used to compare differences between groups at each time point. For imaging data assessed longitudinally, the mixed-models statistical analysis (using the two-way ANOVA test) was performed to compare differences between bleomycin groups at various time points (repeated measures). For the MRI and PET data correlation, simple linear regression was performed, and Spearman’s rank correlation test was used.

All data are expressed as mean values ± standard error of the mean (SEM) unless otherwise specified. *p*-values of less than 0.05 were considered statistically significant. Significance was indicated by ∗ when *p* < 0.05; *p* < 0.01 by ∗∗; *p* < 0.001 by ∗∗∗, and *p* < 0.0001 by ∗∗∗∗, when comparing a bleomycin-exposed group to the corresponding controls. The comparison between various time points of bleomycin-exposed groups was expressed as # when *p* < 0.05; ## when *p* < 0.01; ### when *p* < 0.001; and #### when *p* < 0.0001.

## Results

### Characterization of the model—weights and tissue assessment from terminal samples

**Figure 2:**
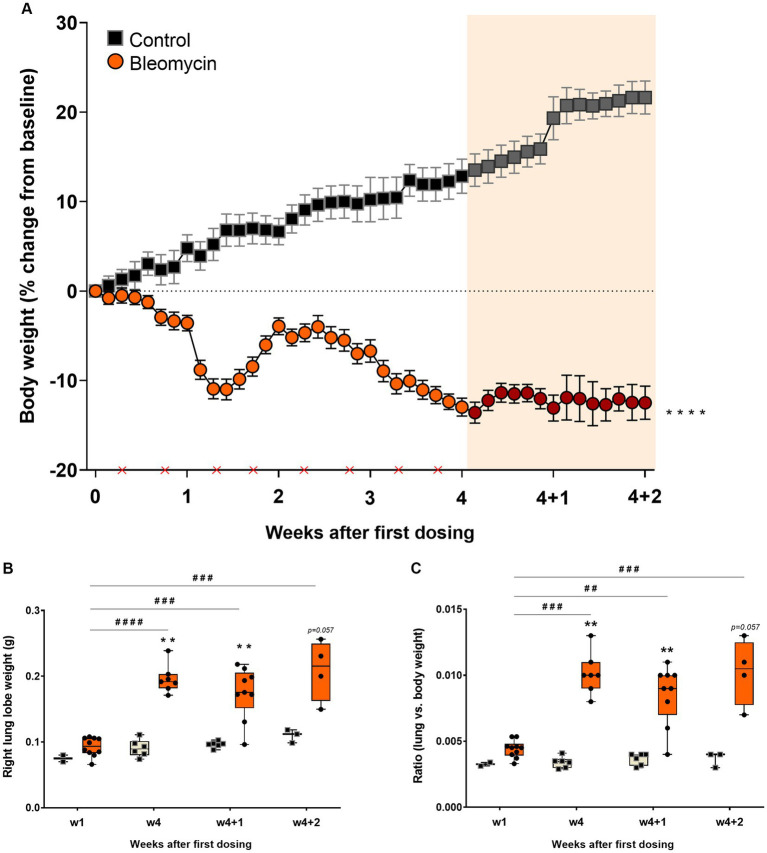
Bleomycin exposure induced body weight decrease and lung weight increase. **(A)** Body weight changes were monitored throughout the whole study. The first 4 weeks included bleomycin exposure (dosing indicated by crosses at the x-axis). The final 2 weeks were resting weeks without dosing, yet a continuous drop in body weight was observed. The resting weeks of longitudinal data points are illustrated by an orange-shaded background in the graph. **(B)** Lung lobe weights were clearly increased in the bleomycin groups during the late phase of the model (w4, w4 + 1, and w4 + 2) and also when compared in terms of lung-to-body weight ratio.

Systemic exposure to bleomycin resulted in decreased body weight, reaching an initial nadir at around 9–10 days after rapid weight loss during the preceding 3 days. The animals recovered during the second week, followed by another decline in body weight that ceased after the last dose of bleomycin (Figure 2A). The body weight of the animals remained stable during the resting period (w4 + 1 and w4 + 2).

The right lung lobes of the bleomycin-exposed animals weighed significantly more compared to the corresponding controls, particularly in the late phase of the model (from week 4 and onward). A significant increase in lung weight was observed over time in animals exposed to bleomycin, comparing the results at 1 week post-exposure to those obtained later in the study (*p* < 0.001 to *p* < 0.0001) (Figure 2B). Even though the bleomycin-exposed animals presented with body weight loss, the lung weights increased. The lung-to-body weight ratio followed a similar pattern observed from the lung weight measures (Figure 2C).

### Histopathological assessment by tissue staining, scoring, and software-based quantification

**Figure 3:**
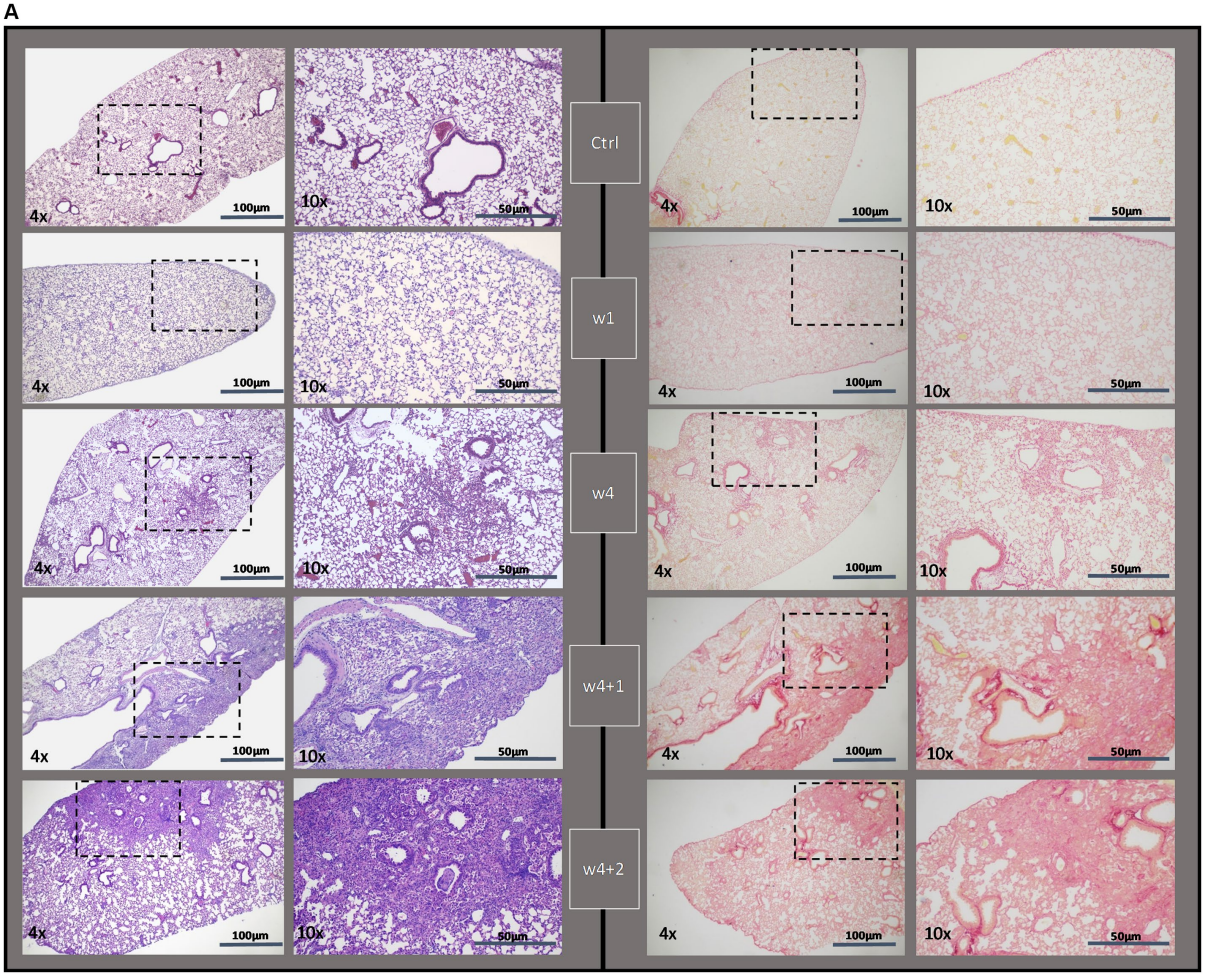

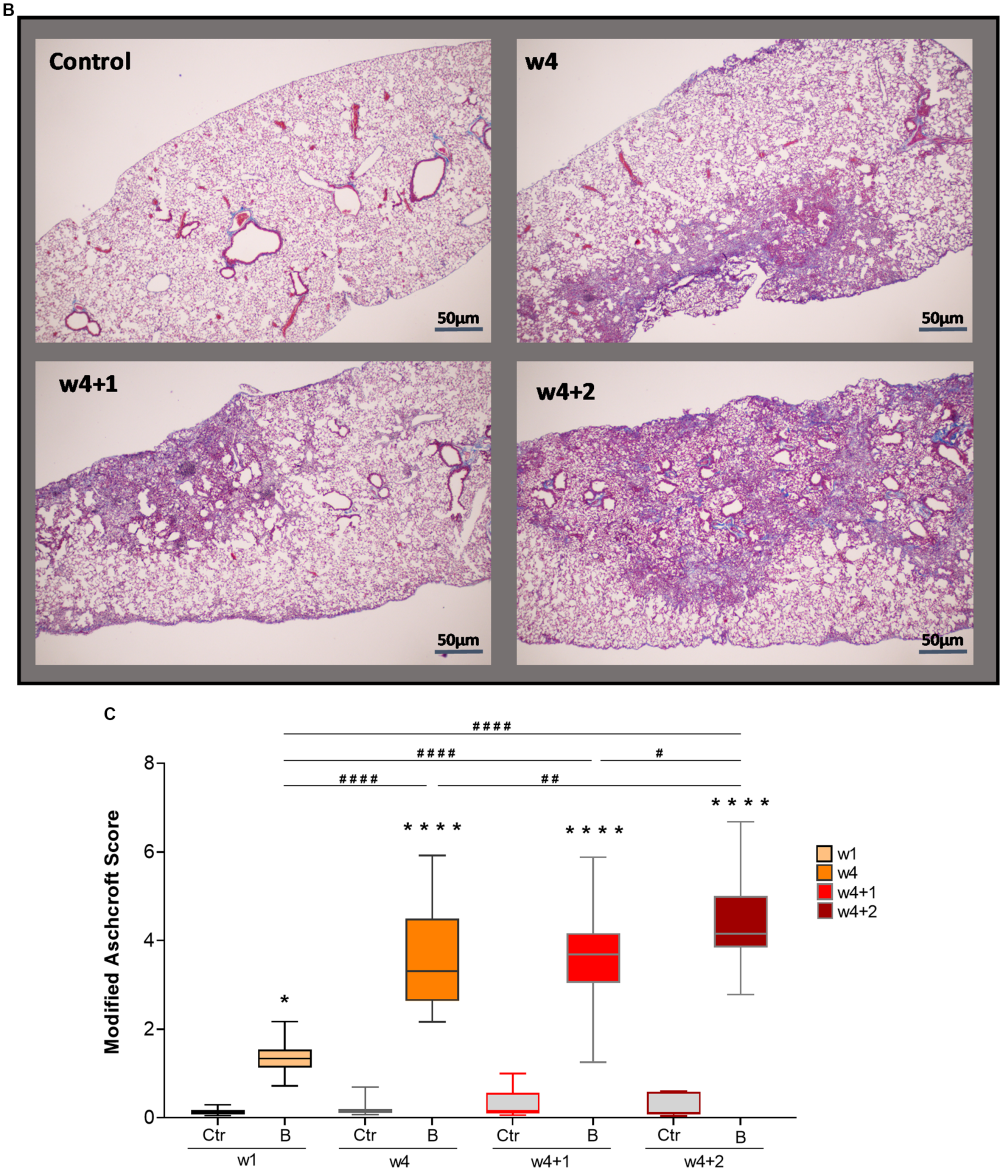
Histologically stained lung sections and fibrosis assessment. **(A)** Representative images from lung tissue sections were stained with H&E and Picro Sirius Red, shown at different termination time points (w1, w4, w4 + 1, and w4 + 2). **(B)** From the Masson’s trichrome stained sections, **(C)** scoring was done using the modified Ashcroft score.

Fibrotic segments were observed during the last week of bleomycin dosing (w4), and disease progression persisted after discontinued bleomycin exposure. Both H&E and Picro Sirius Red stained sections demonstrated fibrotic areas, which continued to increase over time, being particularly evident during the resting period at w4 + 1 and w4 + 2 (Figure 3A). The fibrotic areas were also evident in the Masson’s trichrome stained sections (Figure 3B), used for quantitative assessment of disease by Modified Ashcroft score (Figure 3C). The fibrosis scoring resulted in significantly increased scores in the bleomycin-exposed mice (*p* < 0.0001) compared to control groups (w4 to w4 + 2), as well as significantly increased scores over time, comparing the last week (w4 + 2) toward the earlier time points among the bleomycin groups (*p* < 0.05–0.0001) (Figure 3C). The identified histopathological findings were fibrotic foci, mainly emanating within the subpleural regions, and resembled aspects of IPF and usual interstitial pneumonia (UIP) pathology (24).

**Figure 4:**
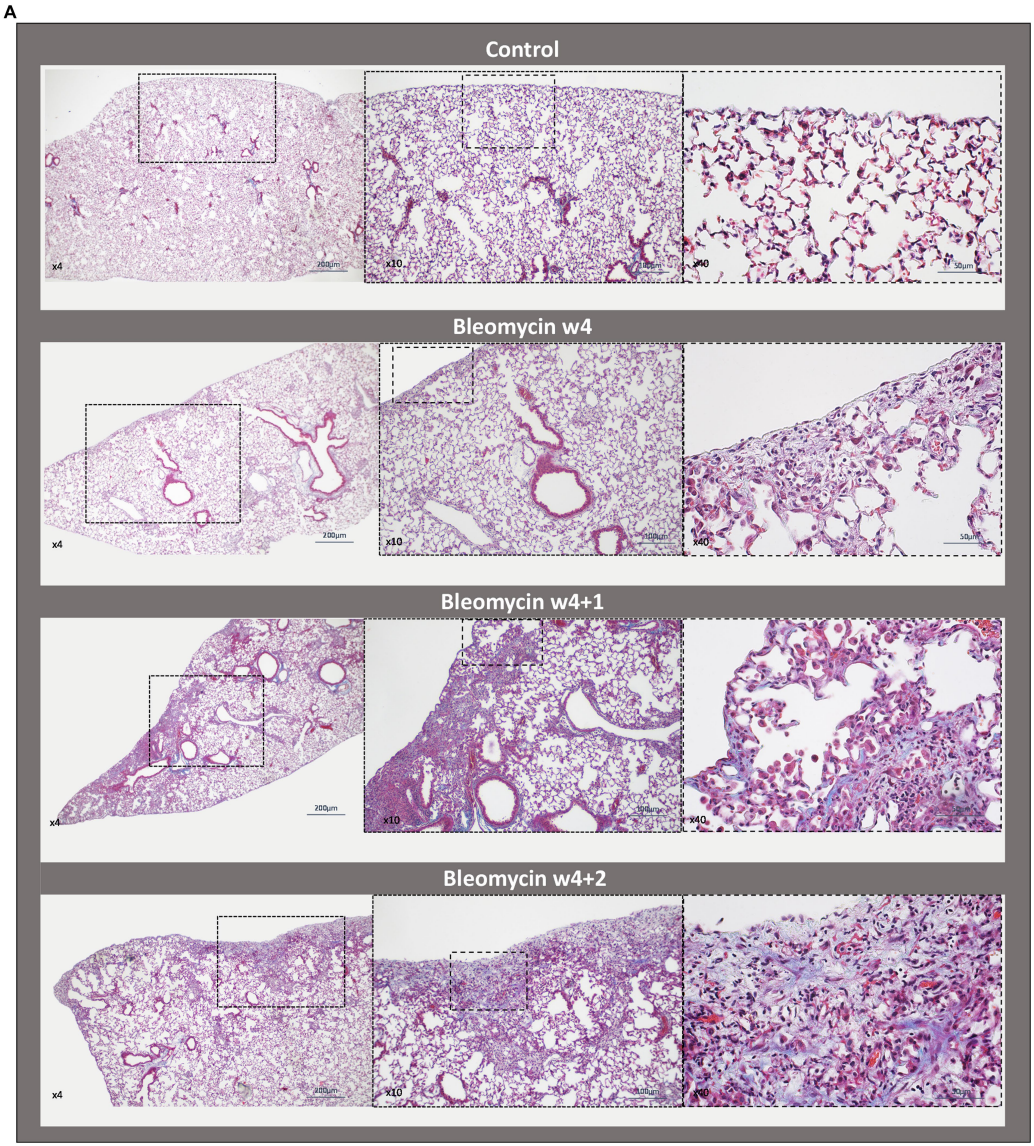

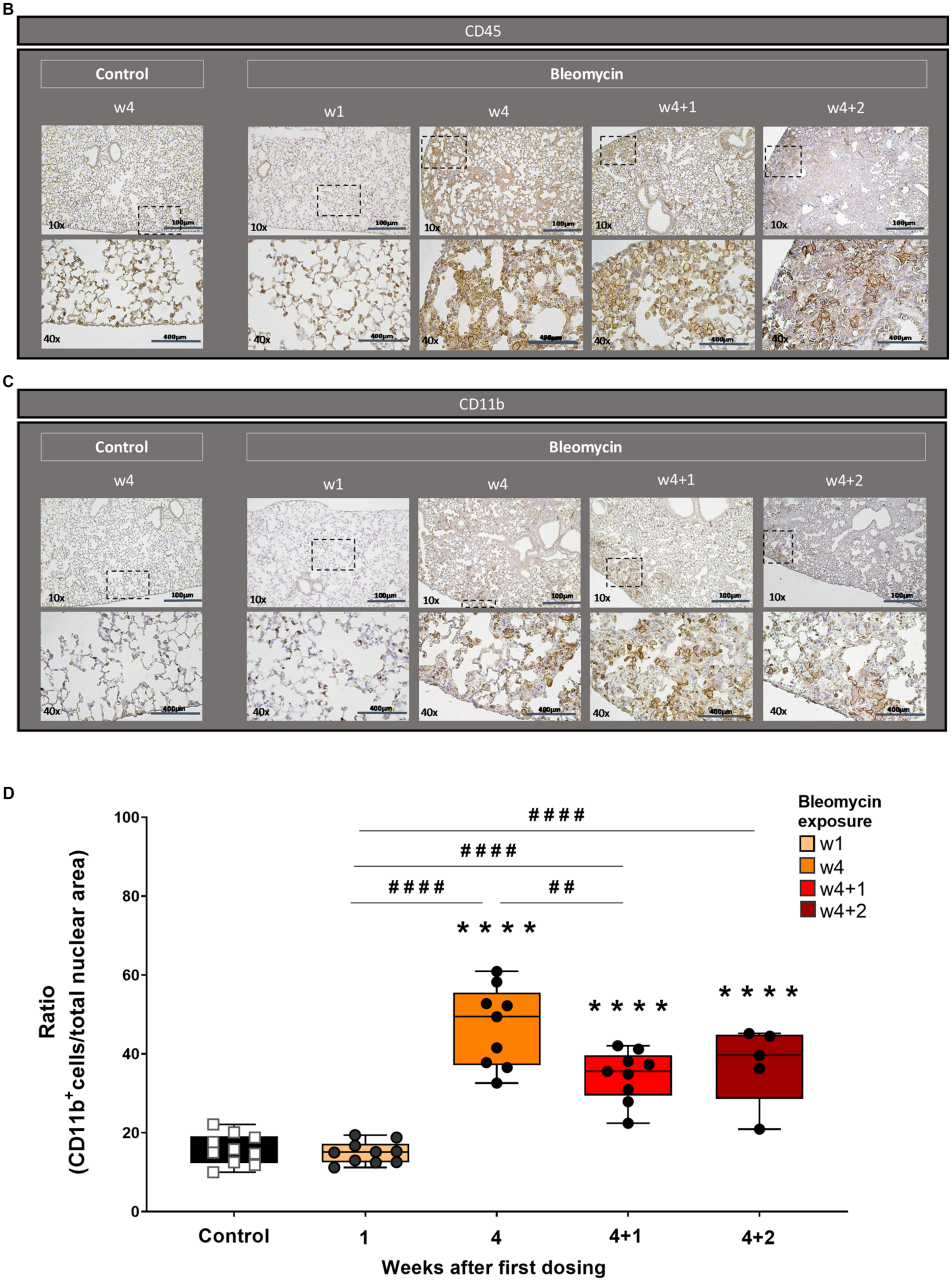
Histopathology and immunohistochemistry of lung sections for inflammation assessment. **(A)** Masson’s trichrome stained lung sections from a control mouse (w4 + 2) lung vs. bleomycin-exposed lungs at time points w4, w4 + 1, and w4 + 2. Inflammatory cells are present as infiltrates during the last dosing and still occurring around the alveolar space as well as within the fibrotic loci at the last time point studied. Representative immunohistochemical staining for **(B)** overall immune cell presence in lung tissue section by targeted CD45 marker and **(C)** specific immune cell targeted marker CD11b, showing the presence of neutrophils and activated granulocytes. **(D)** The CD11b-positive cells were quantified and plotted as mean values for each mouse and presented as box plots with min to max for each time point.

The presence of inflammatory cells infiltrating the alveolar space and also within the fibrotic foci was observed both during the 4 weeks of dosing as well as during the resting weeks of the model (Figure 4A). The immunohistochemistry staining with the pan leucocyte marker CD45 revealed persistent inflammation both during the period of dosing weeks as well as at resting weeks (Figure 4B). The CD11b-positive stained cells (Figure 4C) significantly increased during the last dosing week compared to controls and animals exposed to bleomycin for 1 week (Figure 4D). The quantification of inflammatory cells, as assessed by CD11b-positive stained cells over the total nuclear area, points toward sustained low-grade inflammation over time integrated within the fibrotic lesions in this model.

As bleomycin was administered systemically in this model, other vital organs were also investigated at termination. The histological assessment of the liver and kidneys, after H&E and Picro Sirius Red staining, indicated minor alterations of cells and structures; however, no proper fibrosis was detected in these organs (Supplementary Figures S4A,B).

### The total lung volume and lesion volume assessed by MRI

**Figure 5:**
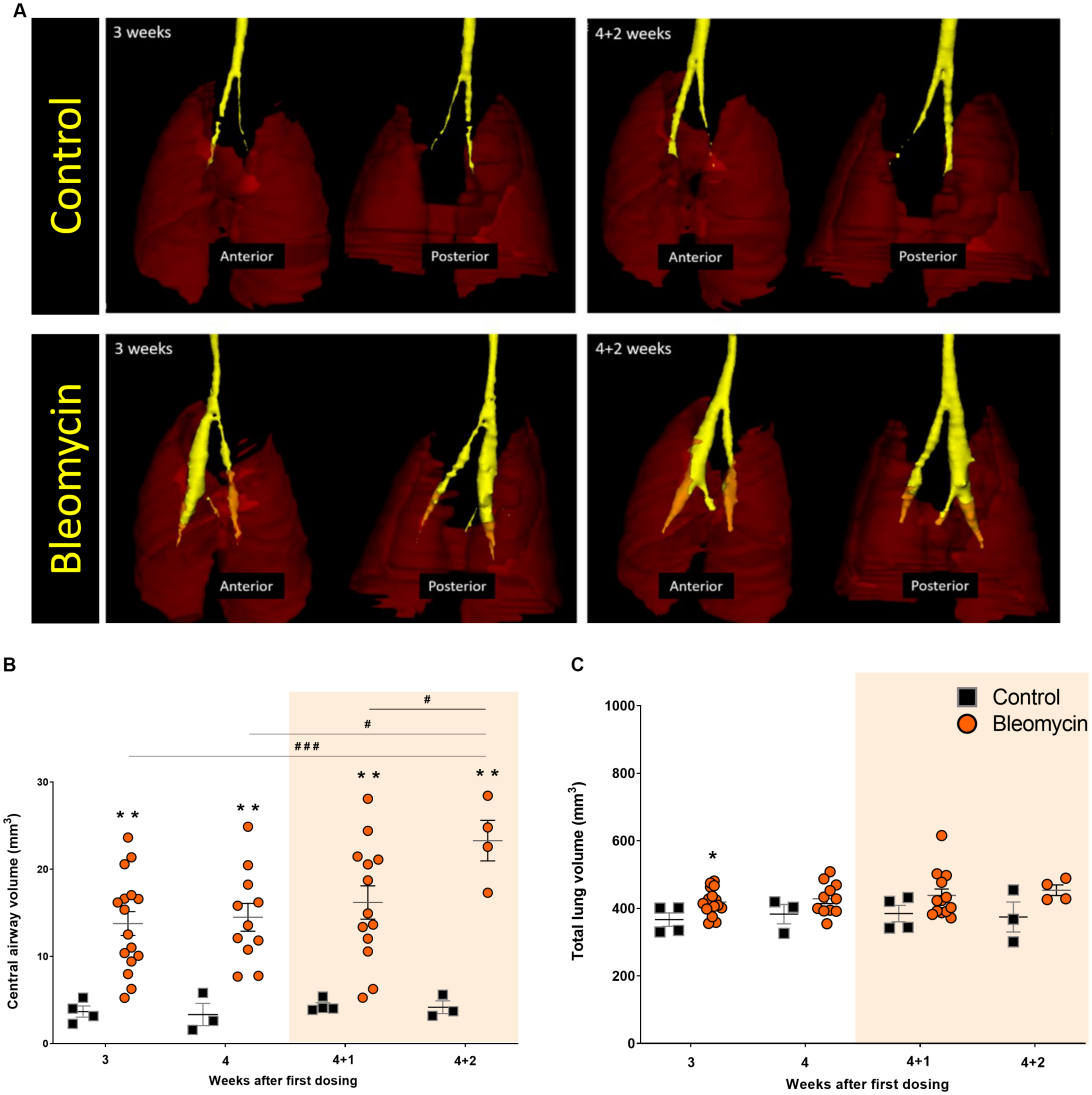
The central airways and total lung volumes assessed by MRI. **(A)** 3D-rendering was performed in MRI-assessed images from a short UTE sequence of 8 μs. The central airways are presented in yellow ROI, while the lungs are shown as transparent dark red ROI. **(B)** The central airway volume was quantified from MRI scans, showing an increase in the bleomycin groups compared to the controls, and over time, changes between the bleomycin groups were also evident. **(C)** The total lung volume was not markedly affected by the repeated bleomycin exposure from w3 to w4 + 2. The resting weeks of longitudinal data points are illustrated by an orange-shaded background in graphs 5B and 5C.

The volume of the central airways was assessed using MRI (Figure 5A). A significantly increased volume of the central airways was observed (*p* < 0.01) for all time points in bleomycin-exposed animals, compared to corresponding controls (Figure 5B). The airway volume was also significantly increased over time (*p* < 0.05–0.001) when comparing bleomycin-exposed animals from the first (w3) and the last imaging sessions (w4 + 2) (Figure 5B). In contrast to the observed dilation of the central airways, the total lung volumes were not markedly affected by bleomycin administration over time (Figure 5C).

**Figure 6:**
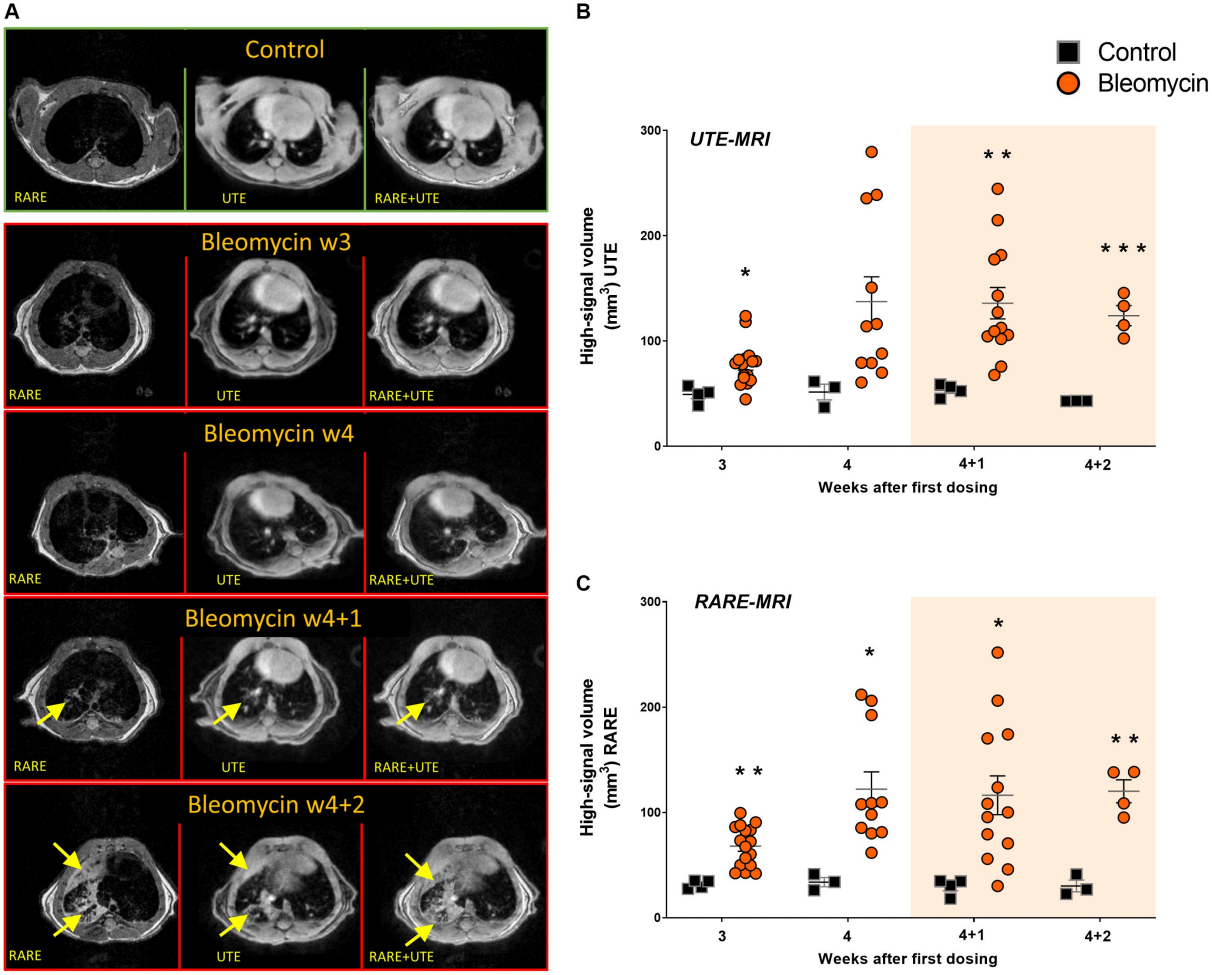
MRI imaging with long UTE and RARE sequences was employed for the assessment of lesion volumes. **(A)** The representative transverse images are shown from the long UTE and the RARE sequence, with visible lesions (yellow arrows) in bleomycin-exposed lungs compared to controls. **(B)** The long UTE sequence scans (1 ms), and **(C)** the RARE sequence scans were used to map lesion volume at different time points during- and after bleomycin administrations in mice, from w3 to w4 + 2. The resting weeks of longitudinal data points are illustrated by an orange-shaded background in graphs 6B and 6C.

Both MRI methods (long UTE and RARE) enabled clear visualization of the lesions in the lungs of bleomycin-exposed animals (Figure 6A). The lesion volume, expressed as a high-signal area, was larger in the bleomycin groups compared to the controls. High-signal voxels representing edema, mucus, or fibrosis were successfully identified by both MRI sequences: long UTE (Figure 6B) and RARE (Figure 6C).

### [^18^F]FDG-signal uptake for tracking metabolism in inflammation and fibrogenesis

**Figure 7:**
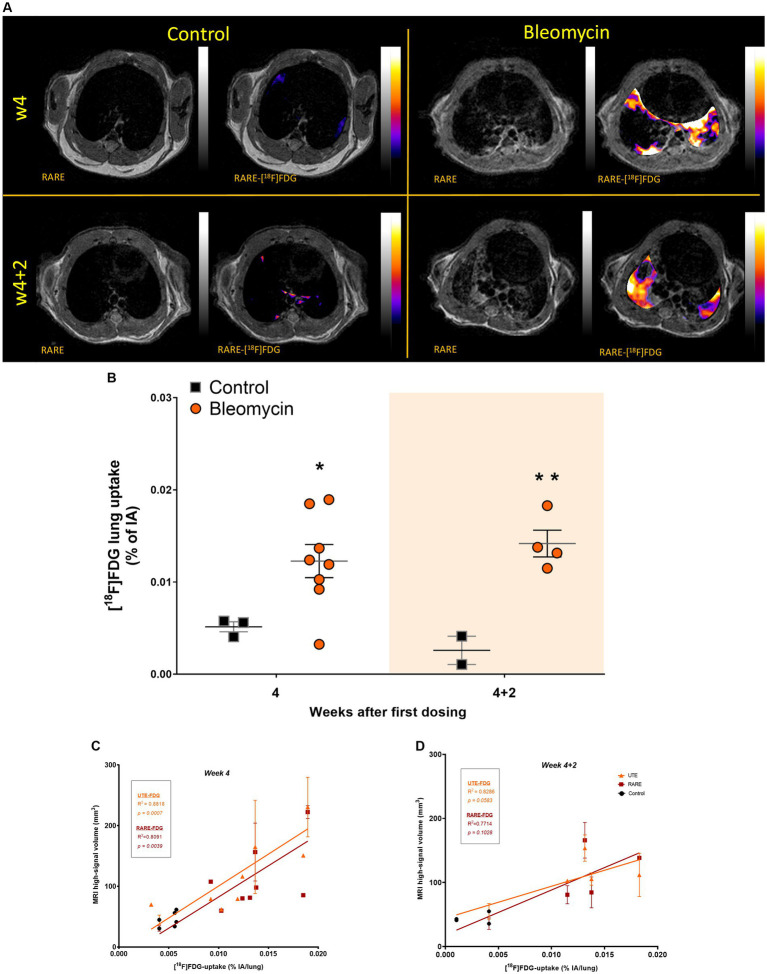
[^18^F]FDG imaging at the last dosing week compared to the last resting week. **(A)** Representative images from [^18^F]FDG imaging, shown at two separate time points. PET images were co-registered and overlaid with MRI for anatomical registration and lesion identification and presented for visualization, only showing PET-signal within the lung-ROI. **(B)** [^18^F]FDG uptake was quantified within the same ROI as MRI, mapping the high-signal areas in the lungs, representing lesions. The resting weeks of longitudinal data points are illustrated by an orange-shaded background in the graph. **(C)** [^18^F]FDG uptake was positively correlated with MRI high-signal volume at w4 and **(D)** at resting week w4 + 2.

PET images were acquired at w4 (last dosing week) and w4 + 2 (2 weeks after dosing cessation) (Figure 7A). There was a significantly higher uptake of [^18^F]FDG in the lungs of bleomycin-exposed animals compared to controls at both time points (p < 0.05), indicating increased metabolism and glucose turnover in bleomycin-exposed lungs (Figure 7B). The [^18^F]FDG uptake within the lung-ROI was correlated with the lesion volumes measured by MRI at w4 (Figure 7C) and w4 + 2 (Figure 7D). This comparison indicates a linear relationship between increasing metabolism along with progressive lesions and/or increasing lesion size.

## Discussion

In this study, we implemented a chronic bleomycin mouse model to study progressive fibrosis using *in vivo* imaging. The animals were given bleomycin (i.p.) twice per week for 4 consecutive weeks. The pathological changes were observed by both MRI and PET imaging during the dosing period, until w4, and in addition, during the 2 resting weeks (w4 + 1 and w4 + 2).

While the lung weights increased in the bleomycin-exposed mice from w4 and onward, the total lung volume assessed by MRI remained fairly constant during this time period. This observation indicated that lung density was increasing. The histological analysis of lung tissue sections demonstrated increased immune cell infiltrates in bleomycin-exposed lungs, which were present not only during the dosing period but also at 2 weeks after the last dosing. Additionally, the specific immunohistochemistry staining for the pan leukocyte marker CD45 (35), targeting immune cells within the tissue, confirmed the presence of inflammation. Moreover, targeting the CD11b marker for specific staining of neutrophils and activated granulocytes (36) revealed significant tissue infiltration at the last dosing week, which was also sustained during the resting weeks. These observations from the histological assessment of lung tissue indicated non-resolving disease with a constant low-grade inflammation present throughout the model, while fibrotic areas increased over time and further escalated during the very last week (w4 + 2) of the model. This increase in fibrotic foci, i.e., extracellular matrix deposition and increased collagen content over time, with the continued presence of inflammatory cells, could explain the increase in lung weights, while lung volumes remained the same.

In clinical practice, non-invasive assessment of lung lesions is mainly performed by HRCT. It is important to consider the limited use of CT for longitudinal assessment of DIILD due to repeated exposures to radiation. Various types of progressive lung diseases are, therefore, increasingly assessed by other modalities besides CT, such as MRI and PET. These modalities are gradually more frequently employed, not only in clinical lung research but also in disease mapping and diagnostics (15, 37, 38). In our study, MRI was used for lung volume assessments and also for exploring and comparing the use of two different MRI sequences, RARE and long UTE, for lesion mapping (32, 39). Using both these sequences, we could identify the so-called “high-signal” areas in the lungs of bleomycin-exposed animals, corresponding to fibrotic and inflammatory lesions. We observed increased high-signal volume in bleomycin-exposed lungs compared to controls already at w4 by both MRI sequences. The high-signal volume was slightly larger when assessed by long UTE sequence compared to RARE. This was probably due to the bright-appearing vessels present in the long UTE images and thus included in the high-signal volume, while vessels essentially appeared as dark voxels in the RARE images and hence were not included in the high-signal volume. In the control animals, the high signal mainly originated from the vasculature, especially in the long UTE scans. Both sequences showed comparable ability to detect lesions within the bleomycin-exposed lungs.

The images acquired by the short UTE MRI sequence with 8 μs echo time allowed for more precise airway rendering, as the normal lung tissue was clearly visible in these images. This property enabled us to measure the airway diameter of the trachea and large bronchi, also referred to as central airway volume, when assessed in 3D. Within these images, a significantly increased airway volume was observed in relation to the corresponding controls at all time points and progressively increased over time compared to the bleomycin-challenged groups. As the bleomycin was administered systemically via the intraperitoneal route, the drug reached the lung tissue via the vasculature and the many capillaries present at a high surface-to-volume ratio in the distal parts of the lung. Thereby, due to the vast capillary bed, the injury caused by bleomycin in this model emanates from the vascular side and progresses from the distal into the central parts of the lung rather than originating from the central airways as in the traditional intratracheal bleomycin model. This was not only evident by the histological assessments throughout the study but also observed using live imaging by MRI and PET. The increased peripheral fibrosis contributing to increased stiffness of the distal regions of the lung is likely to induce traction on the central parts of the lungs, resulting in dilated airways and an increase in central airway volume, as observed by longitudinal imaging in our model. This contrasts with the observations in the acute bleomycin model, where bleomycin is administered as a single intratracheal dose and where a significant increase in total lung volume is generally reported (32, 40, 41). This could imply that, when inducing lesions via the central lung regions, the lung stretches outward to compensate for the decreased functional lung space; thus, increased lung volume is observed. In addition to the significant increase in lung volume observed in previous acute fibrosis models, another non-translational aspect under discussion is the resolution of the disease over time. Animals that survive the single dose instillation of bleomycin and are not excluded later during the experiment due to humane endpoints may resolve and recover from fibrosis (21, 42, 43). Recent publications demonstrated repeated low-dose i.t. administrations of bleomycin for induction of non-resolving fibrosis. Despite achieving sustained fibrosis, the bleomycin in these models reached the lung via local exposure, and the disease etiology and disease pathology do not fully resemble human ILD (44, 45). In our present study, a model with repeated systemic exposures resulted in bronchiectasis-like pathology where the central airways dilated due to fibrosis and stiffness in the distal parts of the lungs, resembling a pathology also known to occur in patients with fibrotic diseases (24, 46, 47). The findings in our model are thus more representative of the human scenario in lung fibrosis, which is described as emerging from the peripheral small airways and progressing further throughout the lung tissue (24).

PET is commonly used in the clinic for metastasis identification in cancer patients, particularly using the tracer [^18^F]FDG (19). Lately, this tracer has become increasingly used also in the assessment of inflammatory diseases and fibrosis assessments (38, 48). Since [^18^F]FDG is a biomarker of increased glucose metabolism, the tracer does not target a specific cell or molecule; thus, it can be used to assess overall disease activity. In our study, the tracer uptake was significantly elevated in the lungs of bleomycin-exposed mice, both within the last dosing week (w4) and during the second resting week (w4 + 2). The increased [^18^F]FDG uptake in the lungs could be an indication of the continuous disease activity in a non-resolving and progressive fibrosis model, both during the dosing period and during the resting period. This is different from what has been shown previously in the acute bleomycin model, with intratracheal administration in rats or mice, where the main peak of lung uptake of [^18^F]FDG occurs in the first week during the inflammatory phase and with a minor increase of [^18^F]FDG observed during the fibrotic phase, 4 weeks after the single dose bleomycin instillation (32, 49). In the clinical setting, it has been shown that increased lung [^18^F]FDG signal is also evident within the fibrotic stage, indicating that this is a valuable biomarker for lung injury in general, both during inflammation and fibrogenesis. (37, 38). Therefore, [^18^F]FDG can be used to evaluate ongoing metabolic processes, regardless of the type of disease profile, provided that [^18^F]FDG levels are elevated above the expected baseline uptake. This tracer is capable of differentiating between healthy and non-healthy states. Consequently, [^18^F]FDG serves as an effective biomarker for detecting active disease progression. As specific fibrosis biomarkers, alternative tracers can be considered, such as those targeting collagen synthesis (CBP) or fibrosis activation (FAP) (50–52). There are studies indicating that the inflammatory cells are the key metabolically active cells; thus, the main energy consumers in lung disease onset (53); however, fibrosis has previously not been considered as a direct energy consuming process in disease, at least not to the same extent as inflammation. In contrast, clinical studies have reported that fibrotic tissues consume glucose to a higher extent than healthy lung tissue regions. Thereby, [^18^F]FDG could be used for disease assessment even when inflammation is not present. This phenomenon, where increased [^18^F]FDG is observed during fibrosis, has been suggested as the induction of glycolysis in the presence of the Warburg effect, even though inflammation is not present (37, 38, 54, 55). Certainly, the increased [^18^F]FDG uptake in our study was observed at two different time points: during the time of systemic exposure to bleomycin and then during the resting period. These results indicate active disease progression at both time points measured and confirm the beneficial potential of using [^18^F]FDG for the assessment of disease activity in patients when mixed pathological profiles might be present, such as fibrosis with inflammatory presence.

Comparing the measures between MRI and PET, a positive correlation was observed between the total [^18^F]FDG uptake and the lesion volume assessed by MRI in the whole lung region.

We used a translational approach to assess a disease model, mimicking the typical human fibrotic disease onset with fibrosis emanating from the distal lung regions and progressing over time. The additional resting weeks post-dosing provided important knowledge to this model as the disease continued to progress, similar to what occurs in patients with DIILD that do not improve after drug withdrawal (2, 46). A further strength of this study was the use of longitudinal multimodality imaging. The information from MRI and PET images were combined to highlight different aspects of the disease model. In addition, the images from the consecutive scan sessions using MRI and PET were in the same frame of reference, as each animal was scanned continuously during anesthesia in a dedicated holder. In this way, the MRI and PET images from the same animal and day could be merged, enabling easier and more accurate image analysis of the lung region. A limitation, though, was that the preclinical PET/CT has limited spatial resolution. Therefore, it was not possible to know if the lesions found by either modality matched exactly geometrically on a single-slice level.

In summary, this study set-up resulted in a translational lung injury model that would be applicable for studies in DIILD, fibrotic diseases, or lung injury modeling overall. The lesions were clearly depicted in the lungs at all measured time points by MRI and PET. In addition, PET tracer uptake correlated positively with the size of the lesions detected by MRI. Histological analysis indicated progressive fibrosis in the lungs of bleomycin-exposed mice, while a low-grade inflammation was present at all time points as fibrosis was observed. Finally, the observed increase in fibrosis, which is known to contribute to scarring and stiffness, developed initially in the distal parts of the lungs and seemed to contribute to the compensatory dilation of the central airways. This is similar to bronchiectasis-like pathology known to appear in the clinical setting post-injury and fibrotic disease (24, 47).

In conclusion, fibrosis development emanated from the peripheral airways while the central airways increasingly dilated over time as the fibrosis burden increased within the lung tissue. These alterations took place while the lung volume was stable over time. Importantly, the approach of repeated and systemically given bleomycin during a 4-week period followed by a 2-week resting period provided a disease development in mice similar to the pathological changes observed in ILD patients overall but particularly relevant for patients exposed to bleomycin, and where drug-induced ILD does not resolve by drug withdrawal is observed.

## Data availability statement

The original contributions presented in the study are included in the article/Supplementary material, further inquiries can be directed to the corresponding author.

## Ethics statement

The animal study was approved by the Animal experiment ethics committee in Lund/Malmö. The study was conducted in accordance with the local legislation and institutional requirements.

## Author contributions

IM: Writing – review & editing, Writing – original draft, Visualization, Validation, Software, Project administration, Methodology, Investigation, Formal analysis, Data curation, Conceptualization. NF: Writing – review & editing, Validation, Software, Project administration, Methodology, Investigation, Formal analysis, Data curation, Conceptualization. JL: Writing – review & editing, Visualization, Validation, Software, Methodology, Investigation, Data curation. Ri: Writing – review & editing, Writing – original draft, Visualization, Validation, Software, Methodology, Investigation, Formal analysis, Data curation, Conceptualization. CC: Writing – review & editing, Validation, Software, Project administration, Methodology, Investigation, Formal analysis, Data curation. AÖ: Writing – review & editing, Validation, Software, Methodology, Investigation, Formal analysis, Data curation, Conceptualization. LO: Writing – review & editing, Writing – original draft, Validation, Supervision, Resources, Funding acquisition, Conceptualization. KW: Funding acquisition, Conceptualization, Writing – review & editing, Writing – original draft, Validation, Supervision, Resources.

## TRISTAN Consortium

TRISTAN-IHI Consortium (#IB4SD-116106) (Translational Imaging in Drug Safety Assessment - Innovative Health Initiative).

## Supplementary material

SUPPLEMENTARY FIGURE S1 Definition of Lung Regions of interest, provided as a reference segmentation done in a healthy mouse lung to create an atlas of how each slice should be segmented, referred to as the template manual.

SUPPLEMENTARY FIGURE S2 a) Longitudinal scans from the same mouse, showing increasing dilation of the central airways, over time (w3 to w4+2). b) Map of the central airways and how the included slices contributed to the total airway volume defined.

SUPPLEMENTARY FIGURE S3 Stained lung sections presented from one control and one bleomycin-exposed lung shown a) with CD11-positive staining (CD11b+) as well as a negative control section (CD11b-) and b) the stained and scanned images with the two step processing involving brightness/contrast adjustment followed by identifying brown vs. blue stained cells assigned mask.

SUPPLEMENTARY FIGURE S4 Liver and kidney histology was assessed after chronic bleomycin exposure. Representative images from tissue sections stained by H&E and Picro Sirius Red, showing minor alteration of morphology yet no progression of fibrosis was observed in neither a) the liver tissue nor in b) the kidneys.
