# Supplementary figures and images for: In vivo MRI and PET imaging in a translational ILD mouse model expressing non-resolving fibrosis and bronchiectasis-like pathology after repeated systemic exposure to bleomycin
Source: Front Med (Lausanne). 2024 Apr 9;11:1276420. doi: 10.3389/fmed.2024.1276420 (PMC11035813; doi:10.3389/fmed.2024.1276420)

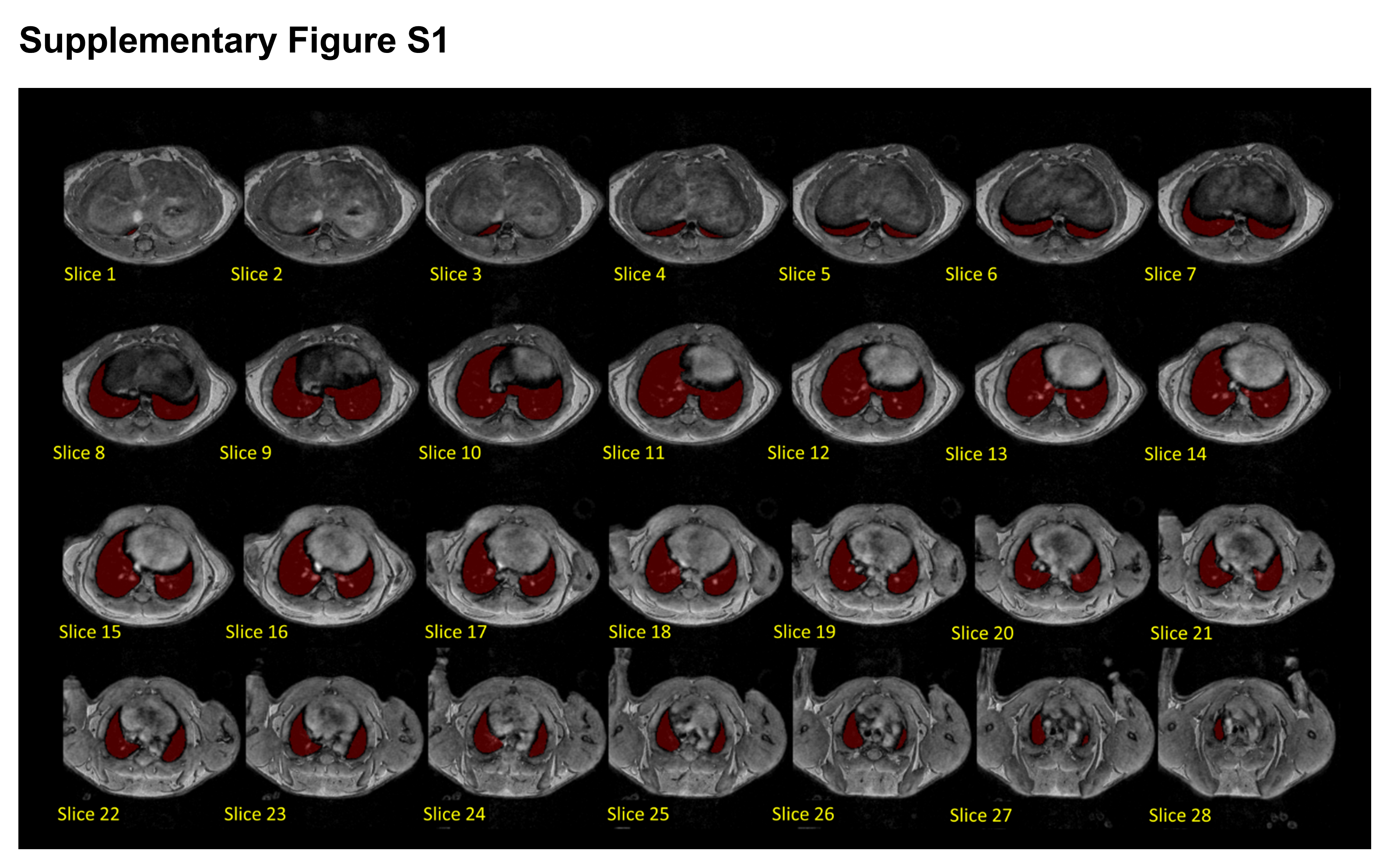

Supplement: SUPPLEMENTARY FIGURE S1 — Definition of Lung Regions of interest, provided as a reference segmentation done in a healthy mouse lung to create an atlas of how each slice should be segmented, referred to as the template manual. [file Image_1.TIF]

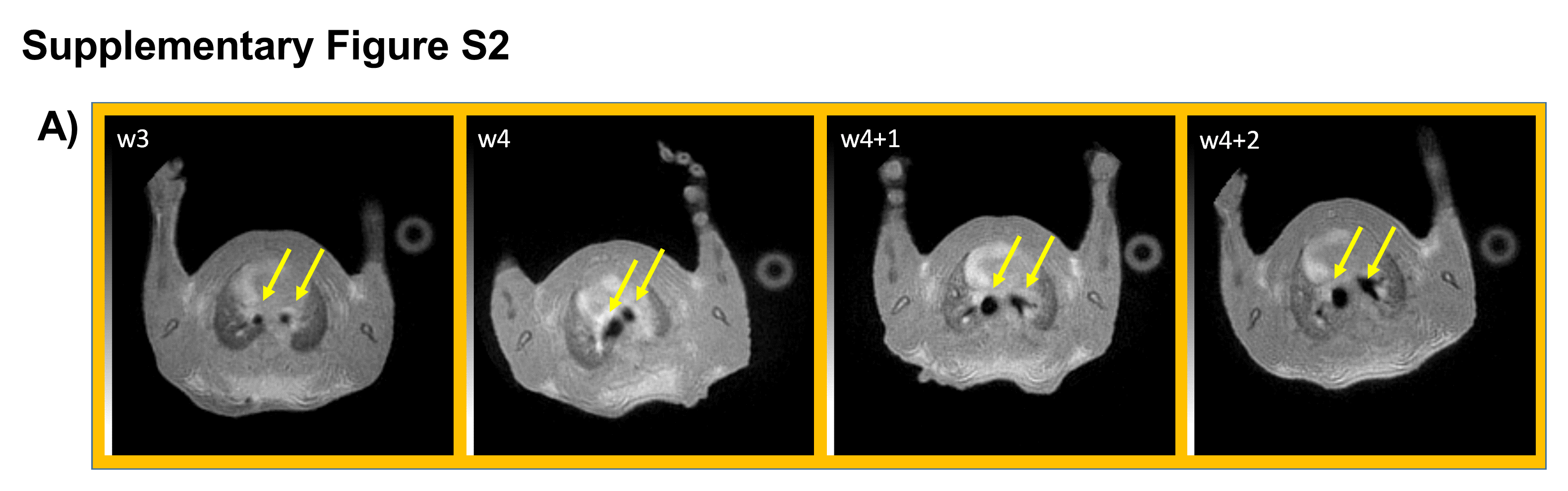

Supplement: SUPPLEMENTARY FIGURE S2 — a) Longitudinal scans from the same mouse, showing increasing dilation of the central airways, over time (w3 to w4+2). b) Map of the central airways and how the included slices contributed to the total airway volume defined. [file Image_2.TIF]

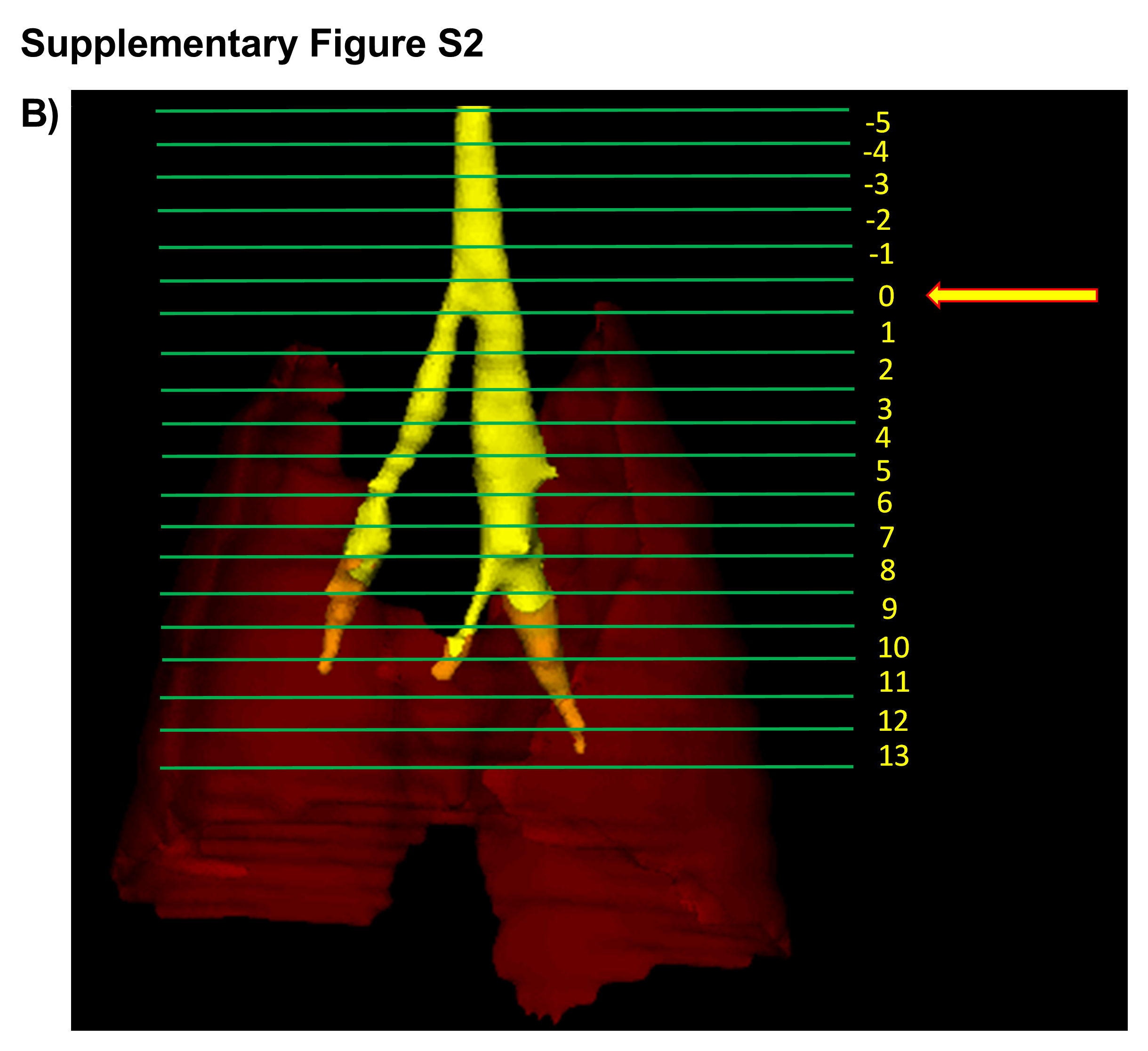

Supplement: SUPPLEMENTARY FIGURE S3 — Stained lung sections presented from one control and one bleomycin-exposed lung shown a) with CD11-positive staining (CD11b+) as well as a negative control section (CD11b-) and b) the stained and scanned images with the two step processing involving brightness/contrast adjustment followed by identifying brown vs. blue stained cells assigned mask. [file Image_3.TIF]

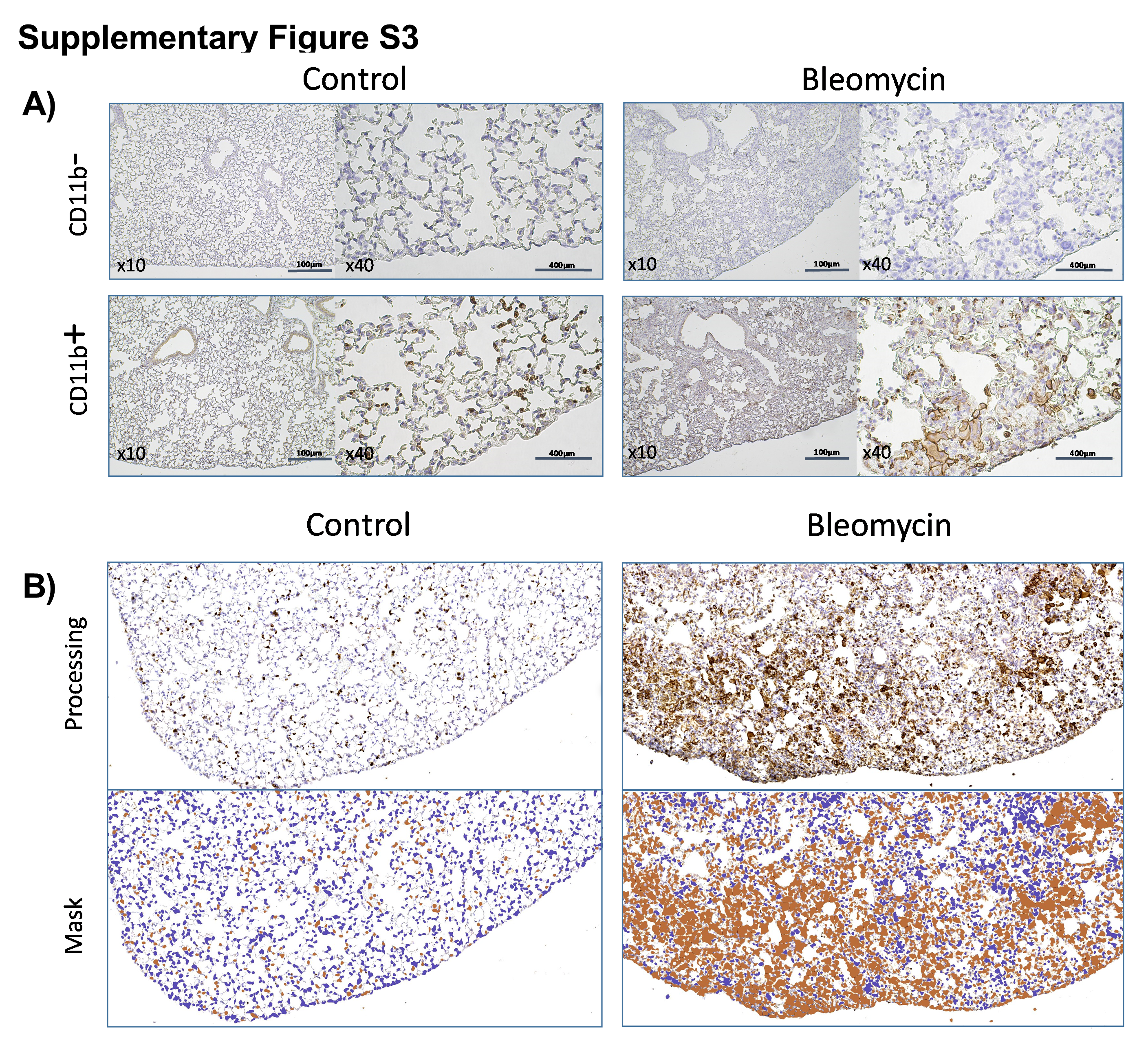

Supplement: SUPPLEMENTARY FIGURE S4 — Liver and kidney histology was assessed after chronic bleomycin exposure. Representative images from tissue sections stained by H&E and Picro Sirius Red, showing minor alteration of morphology yet no progression of fibrosis was observed in neither a) the liver tissue nor in b) the kidneys. [file Image_4.TIF]

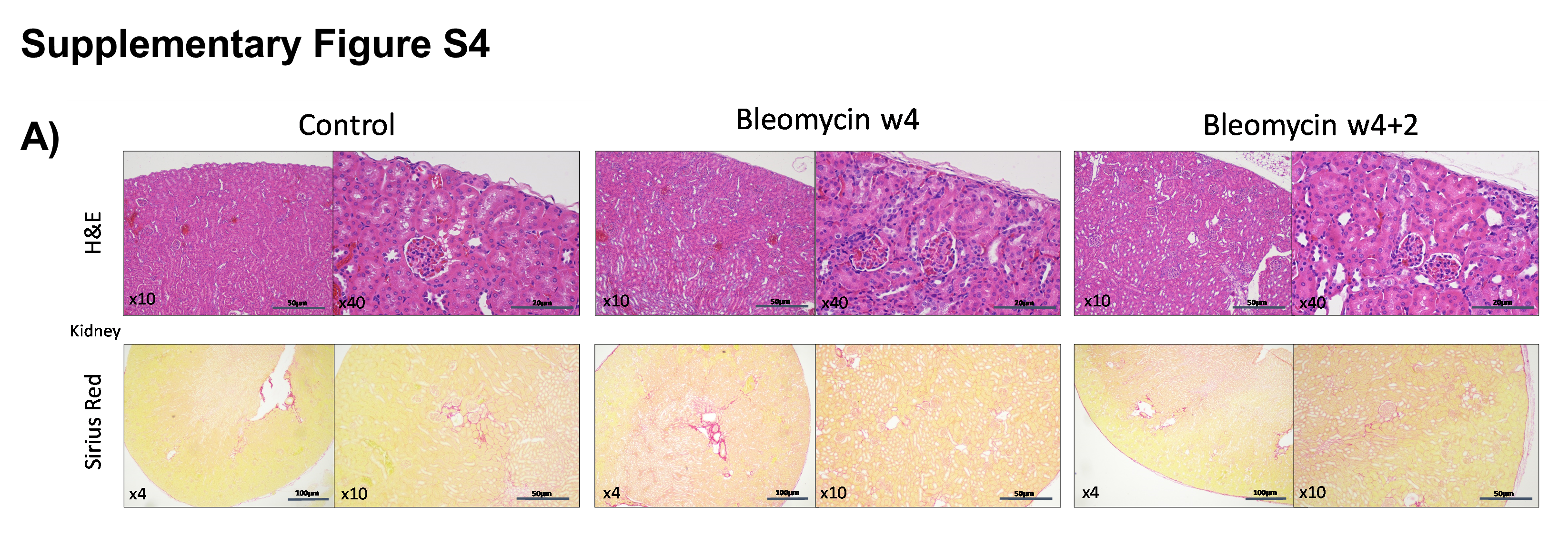

Supplement: Supplementary file 5 [file Image_5.TIF]

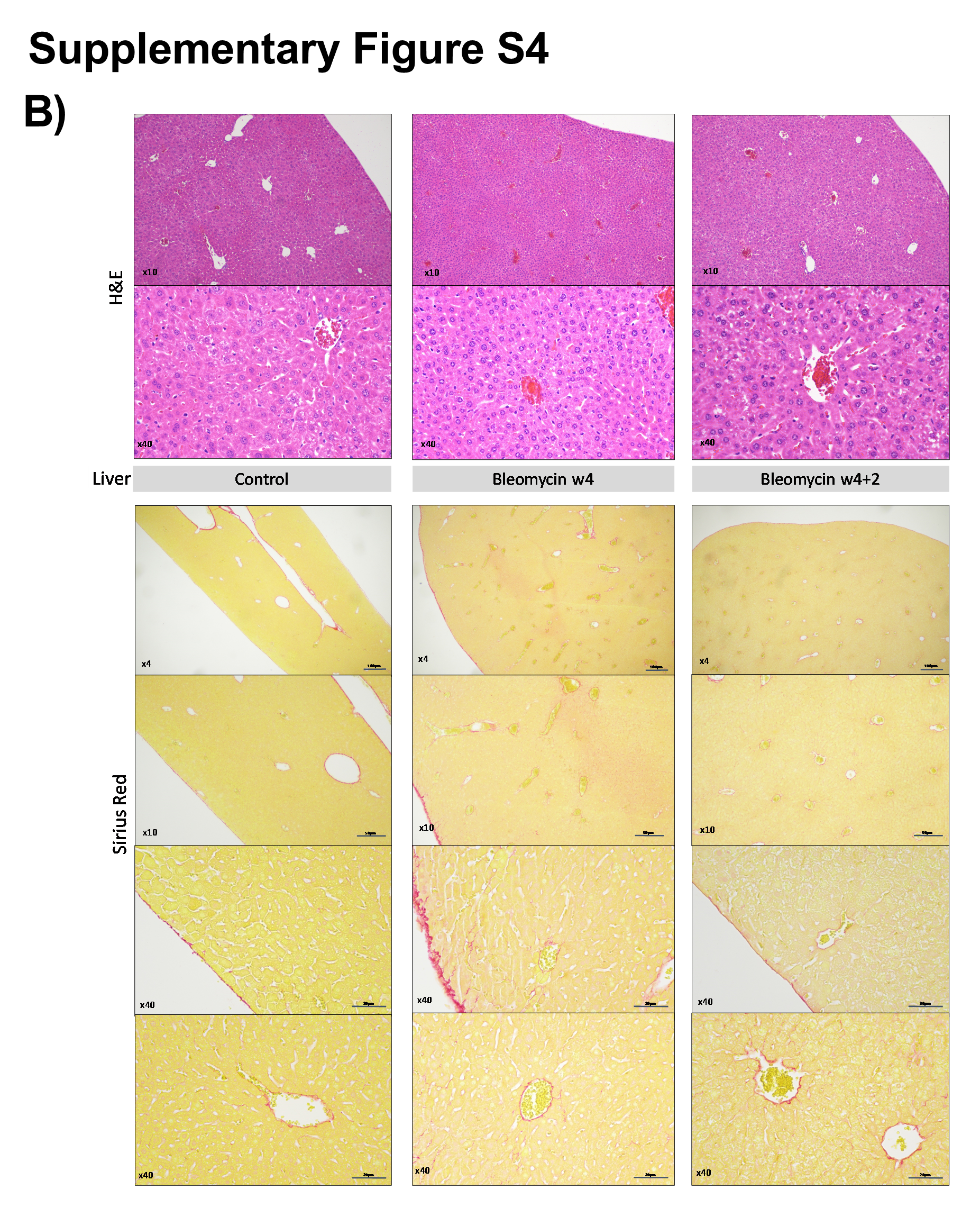

Supplement: Supplementary file 6 [file Image_6.TIF]
